# Creating an Artificial 3-Dimensional Ovarian Follicle Culture System Using a Microfluidic System

**DOI:** 10.3390/mi12030261

**Published:** 2021-03-04

**Authors:** Mae W. Healy, Shelley N. Dolitsky, Maria Villancio-Wolter, Meera Raghavan, Alexandra R. Tillman, Nicole Y. Morgan, Alan H. DeCherney, Solji Park, Erin F. Wolff

**Affiliations:** 1 Program in Reproductive and Adult Endocrinology, Eunice Kennedy Shriver National Institute of Child Health and Human Development, National Institutes of Health, Bethesda, MD 20892, USA; mae.w.healy.mil@mail.mil (M.W.H.); sdolitsky90@gmail.com (S.N.D.); decherna@mail.nih.gov (A.H.D.); dr.wolff@pelexmed.com (E.F.W.); 2 Department of Obstetrics and Gynecology, Walter Reed National Military Medical Center, Bethesda, MD 20889, USA; 3 Trans-NIH Shared Resource on Biomedical Engineering and Physical Science, National Institute of Biomedical Imaging and Bioengineering, National Institutes of Health, Bethesda, MD 20892, USA; mariavwolter@gmail.com (M.V.-W.); meera.raghavan@utah.edu (M.R.); alrotillman@gmail.com (A.R.T.); morgann@mail.nih.gov (N.Y.M.); 4 Pelex, Inc., McLean, VA 22101, USA

**Keywords:** microfluidics, alginate, ovarian follicle, granulosa cell, theca cell, 3D in vitro culture

## Abstract

We hypothesized that the creation of a 3-dimensional ovarian follicle, with embedded granulosa and theca cells, would better mimic the environment necessary to support early oocytes, both structurally and hormonally. Using a microfluidic system with controlled flow rates, 3-dimensional two-layer (core and shell) capsules were created. The core consists of murine granulosa cells in 0.8 mg/mL collagen + 0.05% alginate, while the shell is composed of murine theca cells suspended in 2% alginate. Somatic cell viability tests and hormonal assessments (estradiol, progesterone, and androstenedione) were performed on days 1, 6, 13, 20, and 27. Confocal microscopy confirmed appropriate compartmentalization of fluorescently-labeled murine granulosa cells to the inner capsule and theca cells to the outer shell. Greater than 78% of cells present in capsules were alive up to 27 days after collection. Artificially constructed ovarian follicles exhibited intact endocrine function as evidenced by the production of estradiol, progesterone, and androstenedione. Oocytes from primary and early secondary follicles were successfully encapsulated, which maintained size and cellular compartmentalization. This novel microfluidic system successfully encapsulated oocytes from primary and secondary follicles, recapitulating the two-compartment system necessary for the development of the mammalian oocyte. Importantly, this microfluidic system can be easily adapted for sterile, high throughput applications.

## 1. Introduction

With advances in pediatric cancer therapies, long-term survival has increased to approximately 80% [1]. These patients routinely live into and past their reproductive years, and thus the gonadotoxic effects of these therapies are becoming more clinically relevant. Specifically, cancer survivors experience an increase in premature ovarian insufficiency and shortened reproductive windows [2,3].

Fertility preservation options vary with age, diagnosis, treatment type, and timing of treatment [4], including freezing oocytes, embryos, and ovarian tissue as well as medications before and during chemotherapy to protect the ovaries and the oocytes from the toxic effects [5]. As cryopreservation of embryos or mature oocytes is established and generally safe, it is considered as a reasonable option for sexually mature patients [6]. However, the ovaries of pre-pubertal patients do not respond enough to the exogenous gonadotropin stimulation that is required for controlled ovarian stimulation during oocyte cryopreservation and in vitro fertilization preservation approaches. Thus, current fertility preservation options for young girls are limited to ovarian tissue cryopreservation, which carries concerns for surgical risks associated with the procedure, but mainly the risk of re-implanting malignant cells back into the body [7]. To solve this problem, an in vitro culture (IVC) system for immature follicles and oocytes has been considered as a promising strategy to restore female fertility without the risk of reintroducing tumor cells [8].

Reproductive scientists have made tremendous strides in IVC over the past decade. A dynamic multistep culture system has been designed for ovarian follicular development in mouse [9,10] and cow [11], where the IVC oocytes were fertilized and offspring were successfully born. Recently, second meiotic metaphase oocytes were obtained through multistep in human [12]. Meanwhile, 3-dimensional (3D) culture techniques have utilized a wide range of approaches such as embedding cells in extracellular matrixes [13,14,15], printing 3D scaffolds [16], 3D bioprinting of biocompatible materials and cells [17], decellularization/ recellularization of tissues [18] and also the use of more sophisticated microfluidics technology [19,20].

However, current techniques struggle with consistent maturation of oocytes harvested before the secondary follicular stage [21,22,23,24]. These prior studies focused on creating an artificial ovary, simulating the cortex and medulla with different biomaterials and placing the oocyte granulosa complex within the core of the capsule. Other groups have demonstrated improved growth and survival of earlier stage follicles when co-cultured with ovarian stromal cells [25,26,27,28]. By merging these two approaches, we hypothesized that co-culturing somatic cells within an encapsulated early-stage oocyte would offer better follicular support to the oocyte and improve oocyte development. More specifically, we sought to create a 3D ovarian follicle, with embedded granulosa and theca cells, to better mimic in vivo follicular structure and function.

The primary objective of this study was to construct a microfluidic system to create a hormonally active, artificial, 3D ovarian follicle. We evaluated the consistency of our system, in addition to hormone activity and the long-term viability of the cells within the capsule in long-term culture. The secondary outcome of this study was to place oocytes from early secondary follicles within the core of the capsule.

## 2. Materials and Methods

### 2.1. Design of 3-Dimensional Capsule

**Figure 1:**
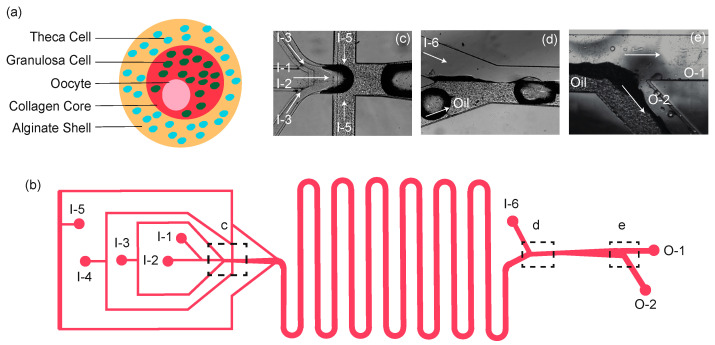
Design of capsule and device. (**a**) A schematic illustration of 3D ovarian follicles. The capsule incorporates murine granulosa cells within a soft collagen core surrounded by a separate layer of murine theca cells suspended in a stiffer alginate shell. (**b**) A schematic view of the microchannel system. Capsules are created at the nozzle where the solutions convene (dotted box, c). Once capsules are generated, they travel through the serpentine channels to allow additional time for gelation and are collected in the top reservoir (O-1). (**c**) A typical image of the boxed region in panel b showing the flow-focusing areas. (**d**) A typical image of the boxed region in panel b showing the entrance of the extraction channel. (**e**) A typical image of the boxed region in panel b showing the exit of the extraction channel. I-1, I-2, I-3, I-4, I-5, and I-6 are the inlets of oocyte, core, shell, Ca-mineral oil emulsion, dispatching, and extraction flows, respectively. O-1 and O-2 are outlets for the aqueous (containing capsules) and oil emulsion flows, respectively.

The capsule design was developed to have spatially distinct core and shell regions. The core consisted of 0.8 mg/mL collagen, 0.05% alginate, and murine granulosa cells suspended within the matrix. The shell consisted of murine theca cells suspended in 2% alginate. Primary oocytes were encapsulated within the core, closely surrounded by theca and granulosa cells (Figure 1a).

### 2.2. Granulosa and Theca Cells

Using the protocol described by Li and Hearn [29], granulosa and theca cells were isolated from the mouse ovaries. Briefly, excised ovaries were dissected in cold Leibovitz’s L-15 medium (Gibco, Thermo Fisher Scientific, Carlsbad, CA, USA) containing 100 mg/mL streptomycin sulfate and 100 U/mL penicillin (Gibco, Thermo Fisher Scientific, Waltham, MA, USA). Granulosa cells (GCs) were collected by gently puncturing follicles from the isolated ovaries with a 29-gauge needle. Residual tissue was reserved for theca cells (TCs) isolation. GCs suspensions were then centrifuged at 400 g for 5 min and resuspended in growth media. The residual ovarian tissue was chopped into approximately 1 mm^3^ fragments and digested with 2 mg/mL collagenase I (Sigma-Aldrich, St. Louis, MO, USA) and 40 U/mL DNase in αMEM (Thermo Fisher Scientific, Waltham, MA, USA) at 37 °C for 30 min followed by neutralization. The enzyme-digested pieces were filtered through a cell strainer with pore size of 70 μm, then centrifuged at 400 g for 5 min and resuspended for purification as described by Magoffin and Erickson [30] with discontinuous Percoll (Sigma-Aldrich, St. Louis, MO, USA). The GCs and TCs were resuspended separately in growth media [MEMα: DMEM/F12 1:1 supplemented with 10 mIU/mL recombinant Follicle-stimulating hormone (rFSH), 3 mg/mL bovine serum albumin, 100 mg/mL streptomycin sulfate, and 100 U/mL penicillin (Thermo Fischer Scientific), 1 mg/mL bovine feutin, 5 μg/mL insulin, 5 μg/mL transferrin and 5 ng/mL selenium (Sigma-Aldrich, St. Louis, MO, USA)] and either used immediately for encapsulation with the microfluidic system or plated and incubated at 37 °C at an atmosphere of 5% CO_2_.

### 2.3. Microfluidic Device Fabrication and Design

**Figure 2:**
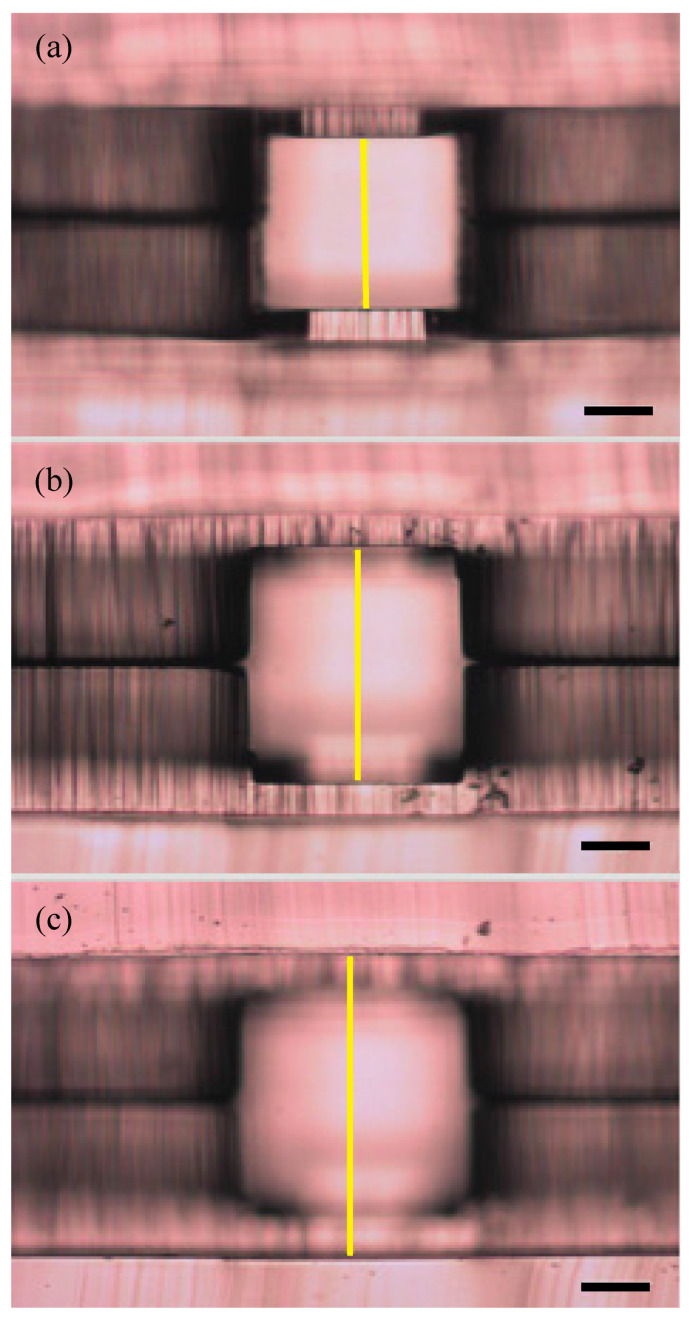
Different nozzle channel heights to ensure correct spatial distribution of core and shell. (**a**) Core channel height, 250 μm; (**b**) shell channel height, 350 μm; (**c**) oil channel height, 450 μm. Yellow line represents the height of each channel. Scale bar, 100 μm.

The microfluidic device design consisted of a three-height nozzle for capsule formation, a serpentine section for alginate gelation, and an extraction channel to transfer the capsules into an aqueous phase (Figure 1b), modified from the design of Choi et al. [31]. To achieve a final polydimethylsiloxane (PDMS) microfluidic device with multiple channel heights (Figure 2), PDMS was molded over a template with corresponding halves of the channel pattern, and the halves were later aligned and bonded together, thus creating the z-symmetry crucial to capsule generation. Templates for the microfluidic devices were patterned onto 4-inch silicon wafers using multilayer photolithography of SU-8 2000 series resists (Kayaku Advanced Materials, formerly MicroChem, Westborough MA), following standard protocols. After cleaning the wafer, each SU-8 layer was deposited using a spin coater (Laurell Technologies, North Wales, PA), and the resist layer underwent softbake, UV exposure with a contact aligner (OAI Model 200, Milpitas CA), and post-exposure bake steps appropriate for the SU-8 thickness. Resist formulation, maximum spin-coating speed, and desired channel height for each of the three patterned layers are detailed in Table 1.

The completed resist pattern was developed in SU-8 developer (Kayaku). The wafers were silanized using tridecafluoroctyltrichlorosilane (UCT Specialties LLC, Bristol, PA, USA) vapor in a vacuum desiccator for an hour. A 4 mm layer of PDMS (Sylgard 184 Silicone Elastomer Kit, Dow Corning, Midland, MI, USA) was poured over the patterned wafer and cured at 80 °C. After demolding, inlet and outlet ports were punched in one of the halves for fluidic access as indicated in the device schematic (Figure 1b). Device halves were then activated by oxygen plasma treatment (PE-100, Plasma Etch, Carson City NV), aligned in the mask aligner, using a thin layer of methanol to prevent premature adhesion, and then brought into contact for bonding. Assembled devices were annealed in an 80 °C oven overnight to strengthen the bond. An additional square of PDMS with two 8 mm diameter holes was then bonded over the two outlet ports to serve as collection reservoirs. To help maintain discrete oil and aqueous flows in the extraction channel, half of the extraction channel was coated with hydrophilic polyvinyl alcohol (Sigma-Aldrich, St. Louis, MO, USA) under laminar flow. The device was then left at 80 °C for 2 days to restore hydrophobicity of the non-polyvinyl alcohol coated PDMS surfaces.

To create the desired core-shell capsule morphology, the shell channel was made taller than the core channel to ensure complete encapsulation of the core solution by the shell solution (Table 1). The oil flow was set such that the aqueous core and shell flows pinched off at the nozzle to form discrete, mono-dispersed capsules (Figure 1c). A separate dispatch flow of calcium-mineral oil emulsion, described in more detail below, was introduced 3.5 mm downstream from the nozzle to help create space between capsules and ensure they gelled separately. The capsules continued the gelation process as they flowed through the serpentine channels to the extraction channel, where capsules in the oil phase met with the aqueous extraction solution (Figure 1d). The completely gelled capsules were drawn into the aqueous phase (Figure 1e) and then flowed into the collection reservoir, which were periodically collected with a pipette. The use of two separate inlet ports to independently adjust the main and dispatch oil flows allowed for better control over capsule size, shape, and spacing. The device operation was monitored with an optical microscope throughout the run.

### 2.4. Encapsulation

Stock solutions of oil/surfactant, shell, low concentration alginate (NovaMatrix, Sandvika, Norway), and extraction solutions were premade and stored for up to a month. The oil/surfactant solution was 95% mineral oil, 4.5% SPAN 80, and 0.5% TWEEN 20 (Sigma-Aldrich, St. Louis, MO, USA), mixed on a stir plate for a minimum of 10 min. The shell solution consisted of 2% alginate and 300 mM d-mannitol in 10mM HEPES (pH 7.2) (Sigma-Aldrich, St. Louis, MO, USA). The low concentration alginate solution consisted of 1% carboxymethyl cellulose (Sigma-Aldrich, St. Louis, MO, USA), 0.5% sodium alginate, and 300 mM d-mannitol in 10 mM HEPES (pH 7.2). The shell and the low concentration alginate solutions were sterile filtered and stored at 4 °C until use. The extraction solution consisted of 6% carboxymethyl cellulose with 300 mM d-mannitol in 10mM HEPES. Due to its viscosity, we were unable to sterilize the extraction solution by filtration, so the solution was passed through a 40 μm cell strainer, autoclaved, and then stored at 4 °C until use.

Immediately prior to each device run, the core solution and the calcium-mineral oil emulsion were prepared. The core solution consisted of 0.8 mg/mL collagen, 0.05% alginate, 0.2% cellulose, and 0.4 mM hydrochloric acid in calcium and magnesium-free PBS. The core and shell solutions were titrated to pH 7.2, and then mixed with the granulosa and theca cells, respectively, under sterile conditions. The calcium-mineral oil emulsion was made by adding 500 μL of 6 M calcium chloride solution dropwise to 2.5 mL of the oil-surfactant mixture under continuous stirring in a 25 mL flask. The emulsion was mixed for an additional 10 min and then used immediately.

The microfluidic device described above was first prefilled with sterile water; syringes with the core, shell, and extraction solutions were placed on syringe pumps, and the inlet tubing was only inserted into the device after the solution had reached the end, to avoid the formation of bubbles. A syringe containing mineral oil and surfactant alone was used for initial device setup to avoid gelation of alginate inside the channels before the flows stabilize. When starting capsule formation, oil/surfactant solution at 900 μL/hr through the main oil channel and 500 μL/hr through the dispatch oil channel. The extraction solution was simultaneously started at 1400 μL/hr. Once a stable interface between the oil and extraction solution was achieved in the extraction channel, pumps for the core and shell solutions were started at 150 μL/hr and 225 μL/hr, respectively. The core solution and shell solution contain approximately 2 and 3 million/mL GCs and TCs, respectively. After the formation of capsules was stabilized, the oil/surfactant solution was replaced by the calcium-mineral oil emulsion. The calcium ions delivered to the capsules from the emulsion caused gelation of the alginate shell as the capsules traveled through the serpentine channel. Once the gelled capsules reached the extraction channel, the hydrophilic capsules were pulled into the aqueous extraction stream from the oil phase ending in the top reservoir. The oil streamed into the bottom reservoir and was removed as waste. Capsules were collected from the top reservoir and placed in a 4-well plate with 10 capsules/well in 500 uL (day 0). 150–200 capsules were collected in approximately 15 min in each run and cultured for further analysis. Starting from day 2, half the media change was performed every other day till day 27.

### 2.5. Spatial Confirmation

To confirm correct compartmentalization within the capsules, green (Bangs Laboratories, IN, USA) and red (Thermo Fischer Scientific, Waltham, MA, USA) fluorescent beads were used in the core and shell solutions, and large nonfluorescent beads (Sigma-Aldrich, St. Louis, MO, USA) were used to simulate the oocytes. Subsequently, granulosa cells and theca cells stained with Cell Tracker were encapsulated to confirm the cells’ correct spatial distribution. Granulosa cells were pre-stained with Cell-Tracker yellow CM-Dil (Thermo Fischer Scientific, Waltham, MA, USA) and the theca cells were pre-stained with Cell-Tracker green CMFDA (Thermo Fischer Scientific, Waltham, MA, USA) following the company’s instruction. Collected microcapsules were imaged using confocal microscopy (Zeiss LSM510413).

### 2.6. Viability for GCs and TCs within Microcapsules (No Oocytes)

On days 1, 6, 13, 20, and 27, GCs and TCs within the microcapsules were tested for viability using LIVE/DEAD^®^ Viability/Cytotoxicity Assay Kit (Thermo Fischer Scientific, Waltham, MA, USA) [32]. Five capsules were chosen at random at each time point to be evaluated. Using Image J (RRID:SCR_003070), the total number of cells per capsule were manually counted by two investigators and averaged. Using the same capsules evaluated for viability, diameters of the capsules were recorded with Image J.

### 2.7. Measurement of Hormones

The hormonal function of the 3-D ovarian follicles encapsulated with GCs and TCs was assessed. Specifically, levels of estradiol, progesterone, and androstenedione in media were measured over 27 days of culture. Estradiol and progesterone levels were measured with competitive immunoassay ELISA kits (Cayman Chemical Company, Ann Arbor, MI, USA) per the manufacturers’ guidelines. Similarly, ELISA was performed for androstenedione levels (MyBioSource, San Diego, CA, USA) with the quantitative sandwich enzyme immunoassay technique. In order to compare hormone function of the 3-D system, 2D cultures of granulosa and theca cells were used as controls. After performing the 3-D runs, the total number of cells within 10 capsules was ascertained by counting the number of cells within each capsule, ×10 capsules, with the confocal microscope. Keeping the ratio of granulosa cells to theca cells consistent to the 3-D group, the 2D control was plated. Starting on day of collection for the 3D wells and day of plating for the 2D controls (day 0), half the media was changed every other day. On day 1, 6, 13, 20, and 27, media was collected and stored at –80 °C until hormone test using the ELISA kits in each batch after all samples were collected. All the assays were processed in triplicate.

### 2.8. Oocyte Isolation and Encapsulation

Oocytes were collected by gently puncturing follicles from the isolated ovaries with a 29-gauge needle. Oocytes from primary and early secondary follicles were identified and collected in Leibovitz’s L-15 medium supplemented with 10% (v/v) FBS and 100 mg/mL streptomycin sulfate and 100 U/mL penicillin [33]. Once collected, they were placed into the microfluidic device for encapsulation via the oocyte channel with a flow rate of 100 uL/hr (Figure 1b). Capsules were placed in growth media [MEMα: DMEM/F12 1:1 supplemented with 10 mIU/mL rFSH, 3 mg/mL bovine serum albumin, 100 mg/mL streptomycin sulfate and 100 U/mL penicillin (Thermo Fischer Scientific), 1 mg/mL bovine feutin, 5 μg/mL insulin, 5 μg/mL transferrin and 5 ng/mL selenium (Sigma-Aldrich, St. Louis, MO, USA)] and half of the media was changed every 2 days. The size and gross appearance of the oocytes were evaluated using a dissecting microscope [34].

### 2.9. Statistical Analysis

Viability and diameter comparisons between days of testing were performed with Kruskal–Wallis test. Hormone analyses 2D versus 3D were compared using Mann–Whitney tests. Results are presented as the mean ± S.D. Results were considered statistically significant when *p* < 0.05.

## 3. Results

### 3.1. Spatial Confirmation

**Figure 3:**
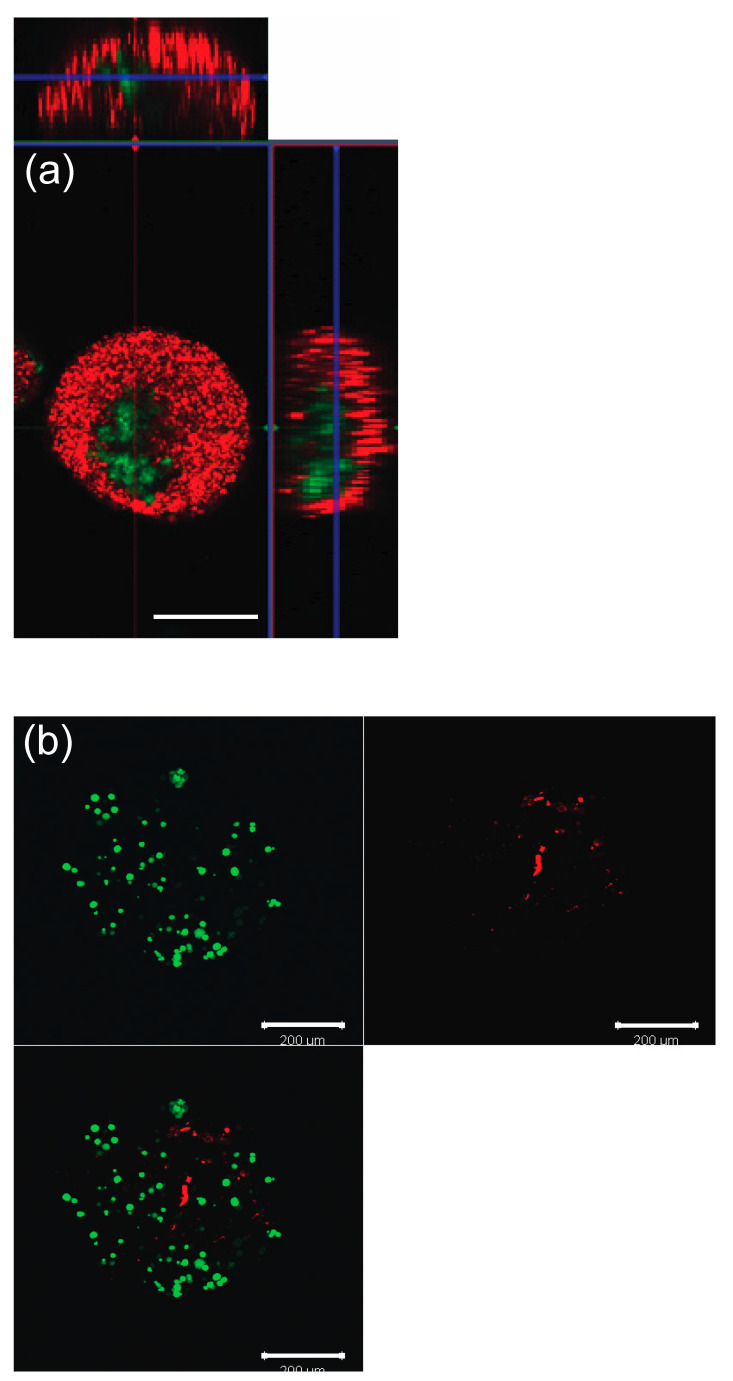
Spatial distribution of capsule (day 0). (**a**) Fluorescent beads (**b**) Granulosa cell and theca cell stained with Cell Tracker to red and green, respectively. Scale bar, 200 μm.

Correct spatial distribution after encapsulation was confirmed both in beads and cells (Figure 3a,b).

### 3.2. Viability Test for GC and TC within Microcapsules (No Oocytes)

**Figure 4:**
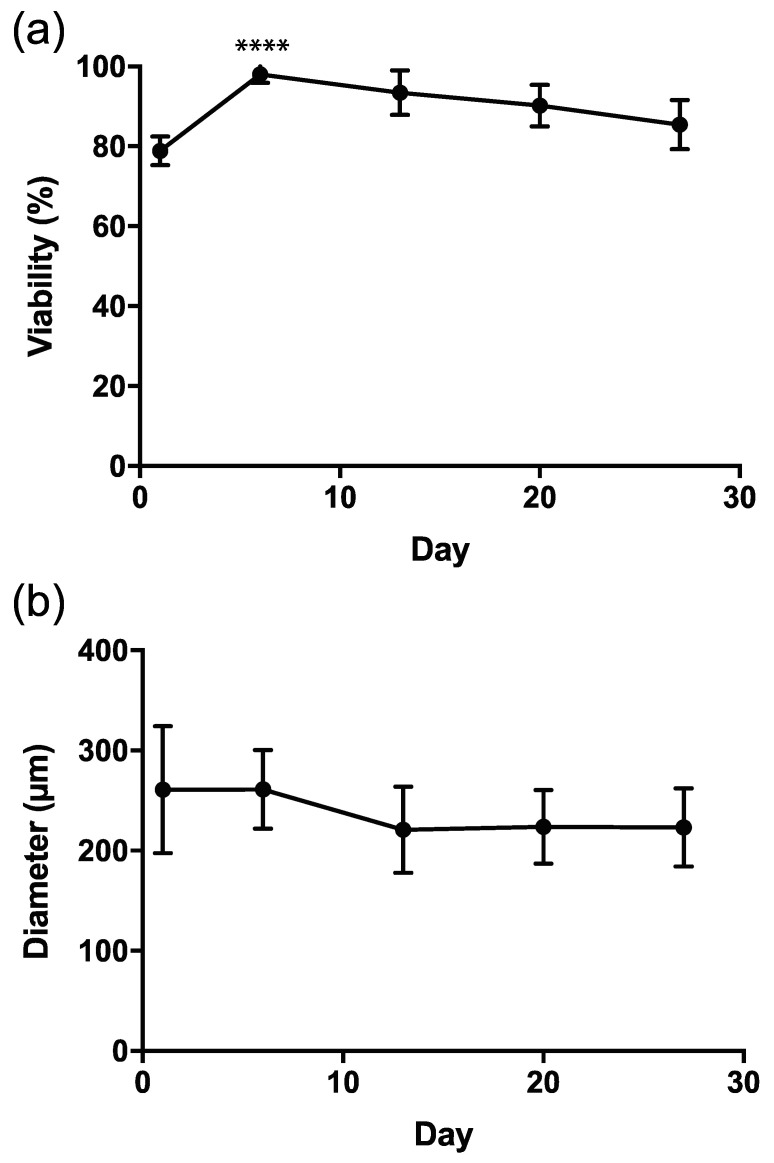
Viability and diameter size of the encapsulated murine granulosa and theca cells over prolonged culture. The figures represent data from four separate experiments. (**a**) viability of encapsulated murine granulosa and theca cells; (**b**) diameter of capsule.

Somatic cell viability was above 78.86% throughout 27 days of culture (Figure 4a). Of note, there was a statistical difference when evaluating days 1 to 6 (*p* < 0.0001) and days 6 to 13 (*p* = 0.0002). No difference was seen between days 13 to 20 and days 20 to 27.

### 3.3. Capsule Size during Culture

The diameters of the capsules were measured. A Kruskal–Wallis test showed no significant change in diameter during 27 days of culture (*p* = 0.216) (Figure 4b).

### 3.4. Measurement of Hormones

**Figure 5:**
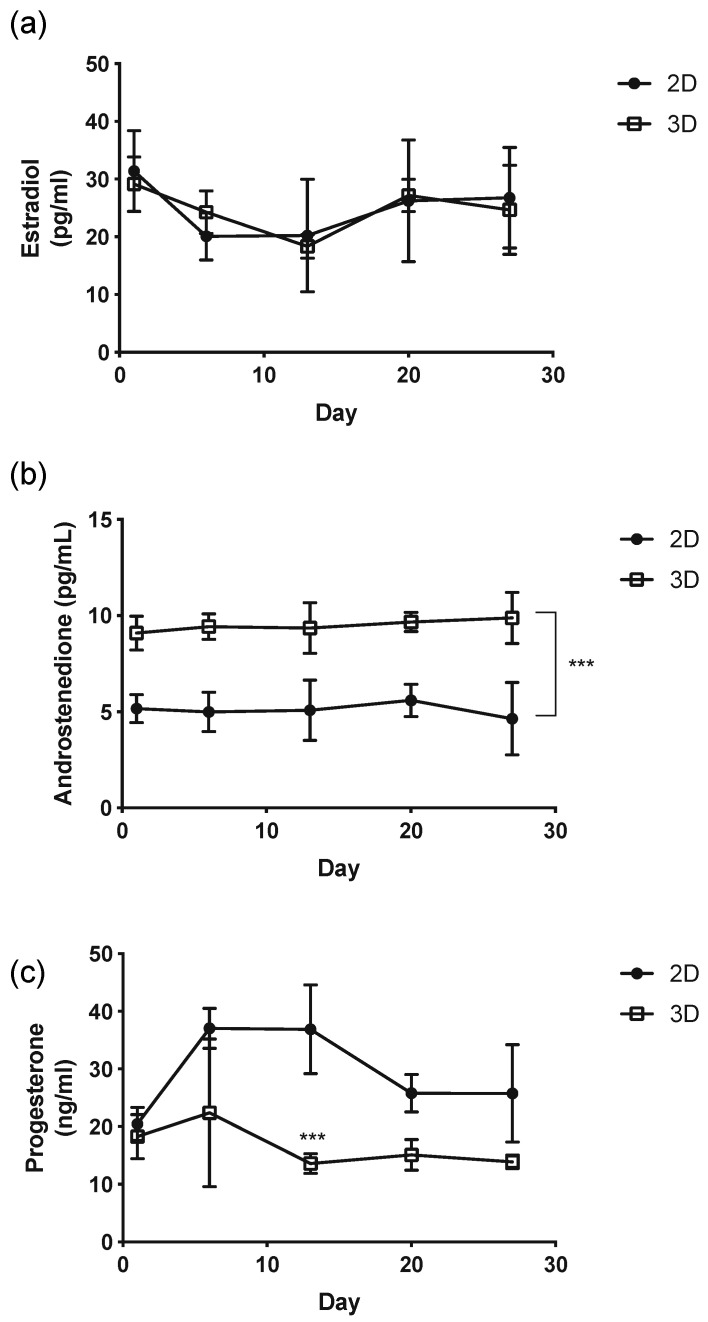
Hormone level as a function of time by co-cultured granulosa cells with theca cells in a 2D and 3D system. (**a**) Estradiol, (**b**) Androstenedione, (**c**) Progesterone. The figures represent data from three separate experiments.

Hormonal assay of culture media revealed preserved endocrine production of estradiol, progesterone, and androstenedione (Figure 5a–c). For estradiol, no significant difference was found between the 2D control compared to the 3D system (Figure 5a). However, for androstenedione, significantly higher levels in the 3D capsules were seen when compared to the 2D control (*p* <0.0003) (Figure 5b). Conversely, for progesterone, significantly higher levels in the 2D control compared to the 3D capsules were found (*p* < 0.0006) (Figure 5c).

### 3.5. Encapsulated Oocytes with GCs and TCs

**Figure 6:**
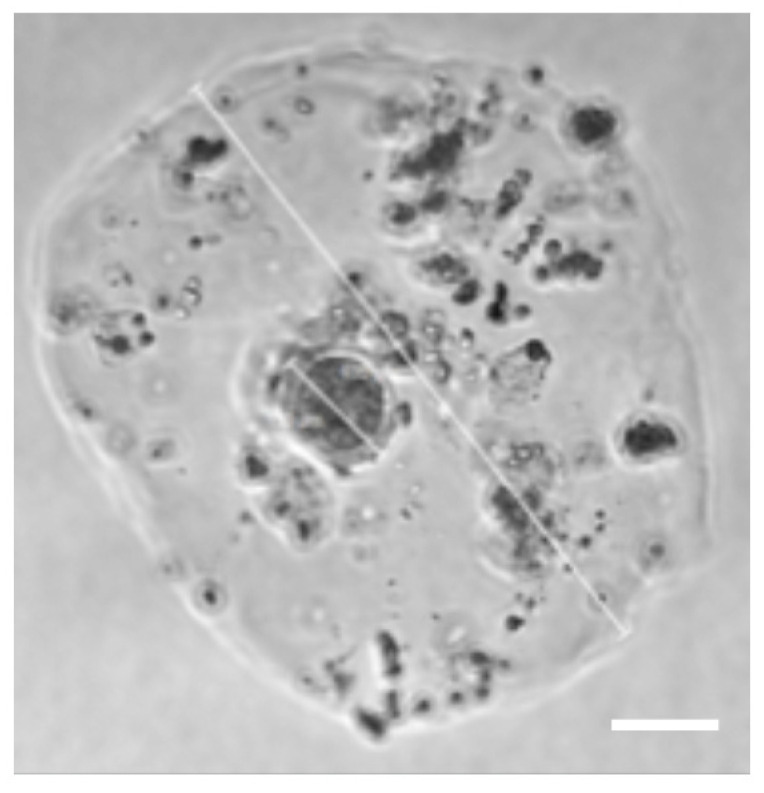
Microscopic image of an oocyte encapsulated with granulosa and theca cells. Scale bar, 100 μm.

**Figure 7:**
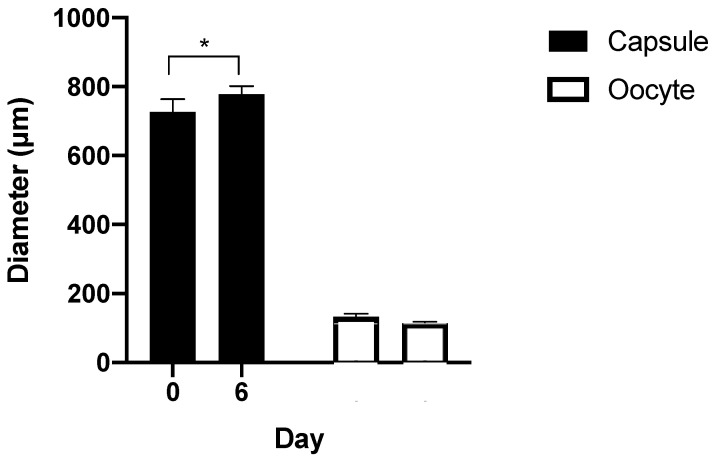
In vitro culture of oocytes in microcapsule for 6 days.

After confirmation of intact endocrine function of GC and TC within the capsules, we introduced oocytes into the system. Initially, we used large nonfluorescent beads similar to the oocyte in size and verified that they situated correctly in the core of the capsule then introduced oocytes. In the first attempts to encapsulate oocytes, we found that the oocytes were very adhesive, making it difficult to get them into the device and leading to bunching. Shortening and priming the inlet tubing for the oocytes overcame this problem. Finally, early secondary follicles were encapsulated (Figure 6). After 6 days of culture, the sizes of the capsules and size of oocyte showed no significant change during culture and no first polar body extrusion observed (Figure 7).

## 4. Discussion

Culturing oocytes in vitro has been an active area of research for several decades; an efficient in vitro culture and maturation system would provide an invaluable research model that could be used to answer fundamental questions of ovarian follicular biology and development. The first successful culture of isolated follicles in vitro was in 1977, which also revealed the importance of granulosa cells for oocyte growth and maturation [35]. Since that time, many advances have been made in in vitro culturing methods, which have improved in vitro follicle and oocyte development.

An important advancement was the transition from 2D (planar, two dimensional) models to 3D models (spatial, three-dimensional) [36,37] which more closely resemble in vivo anatomy. In 2D models, follicular cells and surrounding stroma tend to dissociate and migrate within the plane, thus disrupting essential gap junctions and communications between the oocyte and the granulosa cells that are vital to the growth and maturation of the oocyte [36,38,39]. Overall, the microenvironment of the 2D culture system shows a poor correlation with in vivo conditions [40]. 3D culturing systems have proven to be a more suitable environment for follicular growth, as they allow the ovarian follicles’ morphology, structure, and microenvironment to recapitulate in vivo conditions more closely. While this 3D culture system can overcome the disruption of the follicle architecture and allow cell-cell and cell-matrix interactions within the tissue, implementing the technology consistently has proven more difficult.

Several critical design conditions for these culture systems have been identified [41]. First, culture conditions must be able to preserve oocyte quality [42]. More specifically, temperature control, safe flow rates with correct mechanical design of the system (i.e., no sharp edges to fracture the oocyte during encapsulation), and oocyte-safe solutions should be used in the culture design. Second, the system must support the follicular architecture, but also be expandable to allow for follicular growth [40,41,42,43]. Third, the follicle and the oocyte must be easily retrieved upon at the end of culture. This is not only important for the purposes of studying biological endpoints, but also for the collection of the oocyte for use in clinical care [41]. Fourth, the microenvironment must have the capability to be easily adjustable, in order to have the option to add additional supporting cells to better improve and mimic the ovarian environment [41,44].

A previous successful 3D system that allows for the culture and growth of granulosa cell-oocytes is the alginate hydrogel system [45]. The alginate hydrogel is able to maintain normal follicular architecture and mimic in vivo morphology [46], with a centrally located oocyte and surrounding layers of granulosa and theca cells. Using the inverted drop system to create these capsules, this system was able to support the growth of multilayered secondary follicles isolated from 16-day-old mice. Meiotically competent oocytes were then successfully fertilized and implanted to yield multiple live births in mice [47]. From this method, alginate was shown to be an attractive biomaterial for 3D culture systems because the granulosa and theca cells do not have adhesion receptors for alginate, and therefore, do not interact with the hydrogel [41]. Alginate is often used because it provides mechanical support, maintains cell-cell connections, and is easily degraded by the enzyme alginate lyase, which does not affect the integrity of the follicle [48]. Another group utilized a non-planar microfluidic flow-focusing device to encapsulate early secondary preantral follicles, collected from deer mice, into ~350 μm microcapsules. The microcapsules were composed of an alginate shell and an alginate or collagen core. Choi et al. reported that 28% of the preantral follicles successfully developed into the antral phase with this method, which emphasizes the importance of mechanical heterogeneity [31].

Though In vitro maturation (IVM) has made tremendous strides in maturation of ovarian follicles as early as the secondary stage [21,22,25,28,49,50], no efficient system has been developed to mature primary or primordial stage follicles. It is possible that the secondary oocytes that have been used in other systems have the capability of maturation because they have already developed layers of granulosa and theca cells that are able to support further growth. Thus, we hypothesized that by taking somatic cells and co-culturing them within a 3D capsule with an early-stage oocyte, this system may offer more support transition from the primary oocyte to the secondary oocyte. We sought to first create a scalable system to support this hypothesis. As Sittadjody et al. [51] suggested the importance of spatial arrangement of granulosa and theca cells in order to optimize endocrine function, we encapsulated granulosa, theca cell, and oocyte in core-shell capsule using a non-planar microfluidic flow-focusing device.

The long-term goal for our microfluidic device is to improve human IVM for clinical treatment. Because of the fragility of isolated ovarian follicles, it is important to minimize the time out of culture to optimize viability. Thus, another unique advantage of our design was the ability to encapsulate in one step in a short time. 150–200 capsules were collected in approximately 15 min. We have successfully encapsulated oocytes from early-stage follicles in the core of our 3D capsule, with correctly distributed granulosa and theca cells. With this microfluidic device, uniform capsules were created with easy reproducibility and consistency. Specifically, a uniform distribution of the alginate shell and collagen core was achieved and then maintained over time.

In addition, the ability of the capsules to support not only the viability of granulosa and theca cells, but also hormone activity demonstrating active cellular and endocrine function was integral to our design objective. The somatic cells need to be active in order to support oocyte growth. Over 27 days of culture, the 3D capsules produced estradiol, progesterone, and androstenedione. Using the 2D culture as a control, no significant difference was seen in estradiol activity. The progesterone levels, however, were significantly higher in the 2D culture controls than in the 3D capsule system. Intriguingly androstenedione was significantly higher in the 3D capsule system when compared to the 2D culture controls. 3D capsules may provide enough support for the theca cells to convert progesterone through the steroid pathway more efficiently to downstream androgens (e.g., androstenedione), which can stimulate the hormone production that occurs during normal ovarian follicle growth and development in mammals [52,53]. This demonstrates the capability of these capsules to not only support these cells structurally but also function hormonally.

When oocytes were encapsulated in the core, capsules 2–3 times larger than desired were produced, likely due to the additional fluid required and the size of the oocyte. The larger size of the capsules resulted in less dense GCs and TCs than desired; and likely contributed to the lack of follicular development of the oocytes in culture. Further work on this system to optimize the size of the capsules and adequate number of GC and TCs around the oocyte is required in order to achieve an environment where a primary oocyte can mature in this high throughput system.

## Figures and Tables

**Table 1:** Resist formulation, maximum spin coating speed, and desired channel height for device design.

Title	SU-8 Formulation	Maximum Spin Coating Speed (rpm)	Desired Total Height (μm)	Desired Total Half Height (μm)	Additional Height from Previous Layer (μm)
Adhesion Layer	2000.5–2002	3000	n/a	1.4	n/a
	20% Solids				
Layer 1	2100	2000	250	125	125
(core)					
Layer 2	2050	3250	350	175	50
(shell)					
Layer 3	2050	3250	450	225	50
(oil)					
